# Oxaliplatin-induced peripheral neurotoxicity in colorectal cancer patients: mechanisms, pharmacokinetics and strategies

**DOI:** 10.3389/fphar.2023.1231401

**Published:** 2023-08-01

**Authors:** Fang Cheng, Ruoqi Zhang, Chen Sun, Qian Ran, Cuihan Zhang, Changhong Shen, Ziqing Yao, Miao Wang, Lin Song, Cheng Peng

**Affiliations:** ^1^ State Key Laboratory of Southwestern Chinese Medicine Resources, Chengdu University of Traditional Chinese Medicine, Chengdu, China; ^2^ Department of Pharmacy, Children’s Hospital of Chongqing Medical University, National Clinical Research Center for Child Health and Disorders, Ministry of Education Key Laboratory of Child Development and Disorders, Chongqing Key Laboratory of Pediatrics, Chongqing, China

**Keywords:** oxaliplatin, peripheral neurotoxicity, colorectal cancer, adverse reaction, mechanism, pharmacokinetics, therapeutic strategies

## Abstract

Oxaliplatin-based chemotherapy is a standard treatment approach for colorectal cancer (CRC). However, oxaliplatin-induced peripheral neurotoxicity (OIPN) is a severe dose-limiting clinical problem that might lead to treatment interruption. This neuropathy may be reversible after treatment discontinuation. Its complicated mechanisms are related to DNA damage, dysfunction of voltage-gated ion channels, neuroinflammation, transporters, oxidative stress, and mitochondrial dysfunction, *etc.* Several strategies have been proposed to diminish OIPN without compromising the efficacy of adjuvant therapy, namely, combination with chemoprotectants (such as glutathione, Ca/Mg, ibudilast, duloxetine, *etc.*), chronomodulated infusion, dose reduction, reintroduction of oxaliplatin and topical administration [hepatic arterial infusion chemotherapy (HAIC), pressurized intraperitoneal aerosol chemotherapy (PIPAC), and hyperthermic intraperitoneal chemotherapy (HIPEC)]. This article provides recent updates related to the potential mechanisms, therapeutic strategies in treatment of OIPN, and pharmacokinetics of several methods of oxaliplatin administration in clinical trials.

## 1 Introduction

Over 1.88 million new cases and 915,880 deaths from colorectal cancer (CRC) were estimated in 2020, ranking it the world’s third most commonly diagnosed cancer (after breast and lung cancers) but second in terms of mortality, with 10% incidence and 9.4% mortality, higher than in 2018, according to the GLOBOCAN 2020 estimates of cancer incidence and mortality (Sung et al., 2021; Erratum: Global cancer statistics, 2020; Bray et al., 2018). Developed countries showed the highest incidence of CRC, however recent data revealed a significant increase in CRC cases in heavily populated countries such as China, undergoing rapid economic development (Brody, 2015).

Oxaliplatin, a chemotherapeutic platinum-based agent for the treatment of metastatic CRC (mCRC), was approved by the US Food and Drug Administration in 2004 (De Gramont et al., 2000; Rothenberg et al., 2003; Kuebler et al., 2007; André et al., 2009; Haller et al., 2011). However, oxaliplatin-induced peripheral neurotoxicity (OIPN) is a severe dose-limiting clinical problem that might lead to treatment interruption (Bécouarn et al., 1998; Díaz-Rubio et al., 1998; Lévi et al., 2000; Boku et al., 2007). OIPN was occurs in above 85% patients after treatment of oxaliplatin (Argyriou et al., 2013; Pachman et al., 2015). OIPN represents a clinical adverse reaction that might lead to dose reduction or treatment interruption. The prominent feature of OIPN is the presence of sensory peripheral neuropathy, including dysesthesias, numbness, and sensory loss in a distribution resembling a stocking-and-glove pattern, which is possibly concomitant with neuropathic pain and infrequent motor and/or autonomic damage (Argyriou et al., 2007; Argyriou, 2015; Avan et al., 2015; Staff et al., 2019). Additionally, OIPN has two distinct presentations: a distinctive acute peripheral sensory and motor toxicity which often occurs during or within a few hours after drug infusion. This type of sensory neuropathy is usually rapidly reversible. Alternatively, patients may present with peripheral sensory neuropathy as a result of cumulative dose. These patients further exhibit increases in incidence, intensity, and duration of sensory neuropathy with repeated treatments. This type of sensory neuropathy is moderate and slowly reversible, after treatment discontinuation (Lévi et al., 1992; Hartmann and Lipp, 2003). Oxaliplatin reversible neurotoxicity might result from virtually no accumulation in the plasma (Delord et al., 2003; Merkel et al., 2003), rather than in red blood cells (RBCs) (Gamelin et al., 1997).

Additionally, the oxaliplatin-induced physical damage in multiple ways to lead to functional impairment in neurons including DNA damage, dysfunction of voltage-gated ion channels, transporters, oxidative stress, and mitochondrial dysfunction, *etc.*

Therefore, various strategies are attempted to optimize chemotherapy regimens to prevent and treat OIPN by targeting molecular mechanisms and monitoring pharmacokinetics.

## 2 Oxaliplatin pharmacokinetics and OIPN

The NICE guideline [NG151] recommends that the standard practice for CRC management is appropriate surgery for eligible patients. Therapeutic regimens (FOLFOX and CAPOX) based on oxaliplatin are widely used as first-line treatment in CRC, and as an adjuvant systemic anti-cancer therapy (Table 1) (André et al., 1998; André et al., 1999; Maindrault-Goebel et al., 1999; Maindrault-Goebel et al., 2001; Cassidy et al., 2004; André et al., 2020; Conroy et al., 2021). Common chemotherapy regimens include FOLFOX4, FOLFOX6, mFOLFOX6, and CAPOX. Based on the above-mentioned guideline chemotherapy regimens, various strategies are attempted to optimize chemotherapy regimens to increase antitumor activity but reduce neurotoxicity, including adjustments in dose, duration of infusion, mode of administration, and combination drugs. Of these, the administration of oxaliplatin included intravenous infusion (IV), hepatic arterial infusion chemotherapy (HAIC), pressurized intraperitoneal aerosol chemotherapy (PIPAC), and hyperthermic intraperitoneal chemotherapy (HIPEC). Both antitumor activity and OIPN are closely linked to dose per cycle, cumulative dose, treatment schedule, and duration of infusion. The antitumor activity and OIPN of oxaliplatin and its combined chemotherapy drugs (namely, erlotinib (Van Cutsem et al., 2008), CKD-732 (Shin et al., 2012), OSI-7904L (Clamp et al., 2008), sorafenib (Kupsch et al., 2005), nintedanib (Van Cutsem et al., 2015), bevacizumab (Loupakis et al., 2014), regorafenib (Schultheis et al., 2013), irinotecan (Wasserman et al., 1999; Falcone et al., 2002; Kemeny et al., 2002; Gil-Delgado et al., 2004; Fornaro et al., 2009), capecitabine (Pfeiffer et al., 2006), fluorouracil (5-FU) (Cattel et al., 2003), Ca/Mg (Han et al., 2013), ibudilast (Teng et al., 2020), glutathione (GSH) (Milla et al., 2009) are shown in Table 2. Dosing and pharmacokinetic parameters of total and ultrafiltrate platinum of oxaliplatin are shown in Supplementary Table S1, S2.

**TABLE 1:** Standard adjuvant chemotherapy regimens for CRC in guidelines.

Standard chemotherapy	Time	Oxaliplatin (mg/m^2^)	Leucovorin (mg/m^2^)	Fluorouracil (mg/m^2^)	Others (mg/m^2^)	Duration
FOLFOX 1	day1	infusion 130 2 h	infusion 500 2 h	infusion 1500-2000	-	q2w, 6 months
	day 2	-	infusion 500 2 h	infusion 1500-2000		
FOLFOX 2	day1	infusion 100 2 h	infusion 500 2 h	infusion 1500-2000		
	day 2	-	infusion 500 2 h	infusion 1500-2000		
FOLFOX 3	day1	infusion 85 2 h	infusion 500 2 h	infusion 1500-2000 22 h		
	day 2	-	infusion 500 2 h	infusion 1500-2000 22 h		
FOLFOX 4	day1	infusion 85 2 h	infusion 200 2 h	Bolus 400, infusion 600 22 h		
	day 2	-	infusion 200 2 h	Bolus 400, infusion 600 22 h		
FOLFOX 6	day1	infusion 100 2 h	infusion 400 2 h	Bolus 400 infusion 2400-3000 46 h		
FOLFOX 7	day 1	infusion 130 2 h	infusion 400 2 h	Bolus 400, infusion 2400 46 h		
mFOLFOX 6	day 1	infusion 85 2 h	infusion 400 2 h	Bolus 400, infusion 2400-3000 46 h		
CAPOX	day 1	infusion 130	-	-	capecitabine, 1000 orally twice daily	q3w, 3 months
Single-agent fluoropyrimidine	day 1–14	-	-	-	capecitabine, 1250 orally twice daily	q3w, 6 months
FOLFOXIRI		infusion 85 2 h	infusion 200 2 h	infusion 3200 48 h	Irinotecan, 165 1 h	

**TABLE 2:** Pharmacodynamics of oxaliplatin.

Study	Drug and dose	Population assessable n)	Antitumor activity n)					OIPN (%)	
	(mg/m^2^)		CR	PR	MR	SD	PD	All grades	Grade 3/4
**IV**									
Shirao et al. (2006)	Oxaliplatin 90/130	9	-	-	-	5	4	100	0
Van Cutsem et al. (2008)	Oxaliplatin 130+Erlotinib + capecitabine	18	1	4		11	2	61	9
Shin et al. (2012)	oxaliplatin 130+Capecitabine + CKD732	6	-	-	-	6	-	89	11.1
Clamp et al. (2008)	oxaliplatin 100 + OSI-7904L 6	3	-	-	-	11	1	100	0
	oxaliplatin 130+ OSI-7904L 6	3	-	-	-		1	100	0
	oxaliplatin 130+ OSI-7904L 9	8	-	-	-		-	100	13
Kupsch et al. (2005)	oxaliplatin 130+sorafenib	32		2		17		38	
Van Cutsem et al. (2015)	mFOLFOX6+ nintedanib	85	7	46	-	23	6	78	32
	mFOLFOX6+ bevacizumab	41	4	19	-	15	1	83	29
Loupakis et al. (2014)	FOLFOXIRI + bevacizumab	242	12	152	-	62	16	-	5.2
	FOLFIRI + bevacizumab	245	8	128		82	27	-	0
Schultheis et al. (2013)	FOLFOX + Regorafenib	38	-	4	-	14	-	44	4
	FOLFIRI + Regorafenib		-	3	-	12	-	20	0
Wasserman et al. (1999)	oxaliplatin 85+ irinotecan	24	-	7	-	9	-	-	32
	oxaliplatin 130+ irinotecan								20
Fornaro et al. (2009)	oxaliplatin 85+ irinotecan 165 + capecitabine	15	-	8	-	7	-	73	20
Kemeny et al. (2002)	Oxaliplatin30-50+ irinotecan 40	47	-	12	3	14	18	70	0
	Oxaliplatin 60+ irinotecan 40-75							79	8
Falcone et al. (2002)	Oxaliplatin 100+ irinotecan 175+ leucovorin 200 + 5-FU 3,800	42	5	25	3	8	1	66	5
Pfeiffer et al. (2006)	oxaliplatin 130+ capecitabine 1000	70	-	12	-	36	22	79	6
Cattel et al. (2003)	Oxaliplatin 25/30/35 +5-FU chronomodulated IV	13	-	7	-	4	2	-	15
Han et al. (2013)	oxaliplatin 130+Ca/Mg	19	-	-	-	-	-	84	0
	-Ca/Mg	19						84	0
Teng et al. (2020)	FOLFOX/CapeOx + ibudilast	14	-	-	-	-	-	86	0
	-ibudilast							86	0
Milla et al. (2009)	FOLFOX4 + GSH	14	-	-	-	-	-	100	0
	-GSH	13						69	31
**HAI**									
Kern et al. (2001)	Oxaliplatin 25–150+ leucovorin 200 and 5-FU 600	18	4	6	-	4	4	48	0
Boilève et al. (2020)	Oxaliplatin 100+ leucovorin 5-FU or FOLFIRI + cetuximab/panitumumab or bevacizumab	82	1	36	-	40	-	93	12
**PIPAC**									
Kim et al. (2021)	Oxaliplatin 45/60/90/120/150	16	-	-	-	10	6	-	-

CR, complete response; PR, partial response; SD, stable disease; PD, progressive disease. IV, intravenous infusion; HAIC, hepatic arterial infusion chemotherapy; PIPAC, pressurized intraperitoneal aerosol chemotherapy.

### 2.1 Reversibility of OIPN and pharmacokinetics

No accumulation of ultrafiltrate platinum in plasma may be an important explanation for OIPN reversibility. For the determination of plasma oxaliplatin concentrations, total platinum content is quantified for all platinum complexes, whereas ultrafiltrate platinum quantification considers only platinum complexes not bound to macromolecules. Ultrafiltrate platinum is considered to represent all the antitumor bioactive and toxicity. These are removed from the circulation via irreversible binding to plasma and/or blood components, tissue uptake, and urine elimination. Platinum that is irreversibly combined with plasma proteins or RBCs is believed to have no pharmacological activity (Culy et al., 2000; Graham et al., 2000). Therefore, monitoring platinum in the ultrafiltrate rather than in the plasma is an accepted strategy to control oxaliplatin metabolism. A cumulative pharmacokinetic pattern of oxaliplatin administration (130 mg/m^2^) demonstrated that the platinum concentration showed a high peak 2 h after administration, followed by a rapid decrease (Gamelin et al., 1997). Subsequently, residual levels of total platinum on day 22 were quantified as 0.161 ± 0.045 ug/mL, with significant accumulation in RBCs, with t_1/2_ equivalent to that of RBCs (Koury, 2014), rather than in plasma. The results showed a significant correlation between ultrafiltrate and total platinum concentration curves at all sampling times. In contrast, significant correlation was observed between RBC platinum levels and total platinum at late sampling times (day 8, 15, 22). Another study also reported that platinum accumulation was observed in RBCs except in total plasma or in ultrafiltrate plasma samples (Cho et al., 2006). Thus, OIPN may be reversible after treatment discontinuation.

### 2.2 Oxaliplatin IV and OIPN

IV was the most common mode of administration with high systemic exposure, suggesting a higher incidence of OIPN. The range of oxaliplatin dose administered as IV 2 h was 60–130 mg/m^2^, with 85 and 130 mg/m^2^ being the most frequent doses. Adversely, a single-dose study reported that grade 1 and 2 OIPN were observed in all patients at doses of 90 and 130 mg/m^2^ (Shirao et al., 2006). Furthermore, pharmacokinetic study showed a dose-dependent increase in maximum concentration (C_max_) and exposure/area under the curve (AUC) for ultrafiltrate and total platinum, with approximately 3%–4% of total platinum in ultrafiltrate, and that ultrafiltrate and total platinum were described by a tri-exponential and bi-exponential open model, respectively.

In addition, oxaliplatin accumulation is also a significant contributor to OIPN. The incidence of grade 3 to 4 neurotoxicity increased with cumulative oxaliplatin dose when oxaliplatin (85–110 mg/m^2^) was associated with irinotecan (150–250 mg/m^2^), which was not observed at cumulative doses below 300 mg/m^2^ but presented in 67% of patients receiving above 880 mg/m^2^ oxaliplatin (Wasserman et al., 1999). Another study with patients receiving oxaliplatin (30–60 mg/m^2^) plus irinotecan (40–85 mg/m^2^) reported that 24 out of 49 patients received cumulative doses of oxaliplatin over 1000 mg/m^2^ (Kemeny et al., 2002). In 12% of the subjects, grade 3 neurotoxicity was observed only for cumulative oxaliplatin doses equal or above 1110 mg/m^2^. Grade 2 neurotoxicity was observed in 8 subjects: 6 subjects showed signs of neurotoxicity after receiving a cumulative dose of 960 mg/m^2^ oxaliplatin, whereas 2 patients presented neurotoxicity for cumulative doses of 540 and 720 mg/m^2^, respectively.

Chronotherapy refers to chemotherapy delivery according to 24-h biological rhythms, thus modulating cellular metabolism. Chronotherapy has been proven to be effective in improving drug efficacy and reducing toxicity (Smaaland et al., 1991). There is growing evidence that circadian pharmacokinetics can be transformed into chronotoxicity and chronoefficacy (Dong et al., 2020). Several studies further reported the effect of chronotherapy on oxaliplatin pharmacokinetics. The threshold oxaliplatin concentration (total plasma) at which OIPN was observed in patients submitted to chronomodulated oxaliplatin was 1.50 μg/mL. The correspondent threshold oxaliplatin concentration in ultrafiltrate plasma platinum concentrations was determined as 0.15 μg/mL (Cattel et al., 2003). Kern *et al.* observed that chronomodulated oxaliplatin administration at 20 mg/m^2^ resulted in a higher maximum plasma level of ultrafiltrate platinum at 7 h compared to constant-rate infusion, with similar cumulative renal elimination of platinum in both simulations (Kern et al., 1999). Moreover, possible relationships between pharmacokinetics and patient specific parameters, such as renal function, were conducted by Cattel *et al* (Cattel et al., 2003). Mean total oxaliplatin C_max_ and AUC_tot_ were accumulated, accompanied by decrease of CL and apparent volume of distribution (Vd) from cycle 1 to cycle 6, with steady elimination constant (Ke). Reduction of median AUC_tot_, CL and Vd in ultrafiltrate oxaliplatin over time, might result from changes in Ke or half-life (t_1/2_).

### 2.3 Oxaliplatin HAIC and OIPN

HAIC, a locoregional treatment strategy for hepatic malignancies, consists in a pump or percutaneous port-catheter device surgically implanted into a branch of the hepatic artery. HAIC-based approaches have been used in the treatment of unresectable liver metastases from CRC for decades (Strnad et al., 2021). A retrospective study showed that OIPN was reported in 73.8% of 61 patients, including 9.8% with grade 3 to 4 neurotoxicity (Lim et al., 2017).

A recent study has shown that HAIC-oxaliplatin coupled with systemic chemotherapy and targeted therapy is feasible and safe for CRC patients with unresectable hepatic metastases, allowing resection/ablation in almost 27% of patients (Boilève et al., 2020). Additionally, grade 3/4 toxicities included 40% neutropenia, 43% HAI-related abdominal pain, and 12% neurotoxicity.

Moreover, Lévi *et al.* suggested that systemic drug exposure helped explain OIPN for HAIC oxaliplatin, possibly related to a slightly reduced systemic drug availability and higher availability at the liver organ during HAI as compared to IV (Lévi et al., 2017). Kern *et al.* reported ultrafiltrate platinum AUC increased linearly with increasing dose in oxaliplatin HAIC administration (Kern et al., 2001). A reduction of AUC and Vd was observed for oxaliplatin HAIC 135 mg/m^2^ for 4 h compared to IV 130 mg/m^2^ for 4 h (Kern et al., 1999).

### 2.4 Oxaliplatin PIPAC and OIPN

PIPAC, a novel laparoscopic intraperitoneal chemotherapy delivery technique, improves the distribution and tissue penetration of chemotherapeutic drugs used to treat peritoneal metastases. Repeated PIPAC with oxaliplatin appears to be a safe, feasible, and well-tolerated therapy with reduced toxicity, high intraperitoneal concentration, and low systemic concentration (Demtröder et al., 2016; Rovers et al., 2019). The pharmacokinetic study showed a linear response between dose, C_max_, and AUC, indicating that systemic oxaliplatin exposure was enhanced with growing PIPAC dosing (Kim et al., 2021). The platinum level in the ultrafiltrate was calculated as 11% of that in total platinum at PIPAC oxaliplatin 120 mg/m^2^. Systemic platinum exposure at 120 mg/m^2^ was 3.8% of that reported for IV for 2 h of single-dose oxaliplatin at 130 mg/m^2^ (Shirao et al., 2006). Additionally, OIPN was not observed in 16 patients. Another pharmacokinetic study reported that oxaliplatin concentrations were 3- to 4-times higher in tissue exposed to aerosol than in unexposed muscle at a dose of 90 mg/m^2^ (Dumont et al., 2020). Overall safety showed Grade 1 to 2 neurotoxicity occurred in 4 out of 19 PIPAC sessions during PIPAC with oxaliplatin 90 mg/m^2^; and grade 1 to 2 and grade 3 to 4 neurotoxicity occurred in 1 of 13 PIPAC sessions during PIPAC at a dose of 140 mg/m^2^, respectively.

Electrostatic PIPAC (ePIPAC) had higher tissue penetration of the chemotherapeutic drugs compared to PIPAC due to addition of electrostatic precipitation into the aerosol (Kakchekeeva et al., 2016). Lurvink *et al.* described that ultrafiltrate platinum AUC after ePIPAC was similar to that of after IV oxaliplatin at 90 mg/m^2^, and higher than that of PIPAC (Lurvink et al., 2021). Urine concentrations of oxaliplatin declined rapidly, and no oxaliplatin accumulation was detected between the various ePIPAC procedures. Unfortunately, the adverse effects are not described.

### 2.5 Oxaliplatin of HIPEC and OIPN

Peritoneal carcinomatosis (PC) is a general event in the natural history of CRC. A promising therapeutic option is cytoreductive surgery plus hyperthermic intraperitoneal chemotherapy (HIPEC, also known as IPCH) for patients with isolated, resectable PC, capable of increasing median survival to approximately 63 months with a 5-year survival rate of 51% (Elias et al., 2009).

The pharmacokinetics of HIPEC with oxaliplatin after complete cytoreductive surgery indicated that peritoneal oxaliplatin concentration was 25-times higher than that in the plasma at 460 mg/m^2^, whereas AUC was lower than that previously reported for IV oxaliplatin (130 mg/m^2^) (Elias et al., 2002). Oxaliplatin penetration was 17.8 times greater in the tumor than in non-bathed tissues. Moreover, the authors also failed to identify any serious hematological, renal, or neurological toxicities, except for 2 fistulas and 3 deep abscesses (Elias et al., 2002). Subsequently, the authors revealed that an additional combination of intraperitoneal irinotecan (400 mg/m^2^) on the above regimen resulted in 2.5% hospital mortality, 25% non-hematological complication even 58% grade 3–4 hematological adverse effects (Elias et al., 2004). Similarly, Quenet *et al.* came to the same result: there was no advantage to intensifying HIPEC with the addition of irinotecan, as opposed to the results of intravenous combination therapy (Quenet et al., 2011). However, the most common grade 3–4 side effects with HIPEC were haemorrhage, digestive leakage, and haematological adverse events, and no neurotoxicity appeared to be observed (Goéré et al., 2020; Quénet et al., 2021). However, a retrospective study suggested that postoperative oxaliplatin-based HIPEC might contribute to improve ascites-free survival, but is accompanied by high neurotoxicity (Sun et al., 2021). Therefore, it requires further studies with large samples to observe the antitumor activity and OIPN of HIPEC oxaliplatin.

## 3 Molecular mechanisms

**FIGURE 1:**
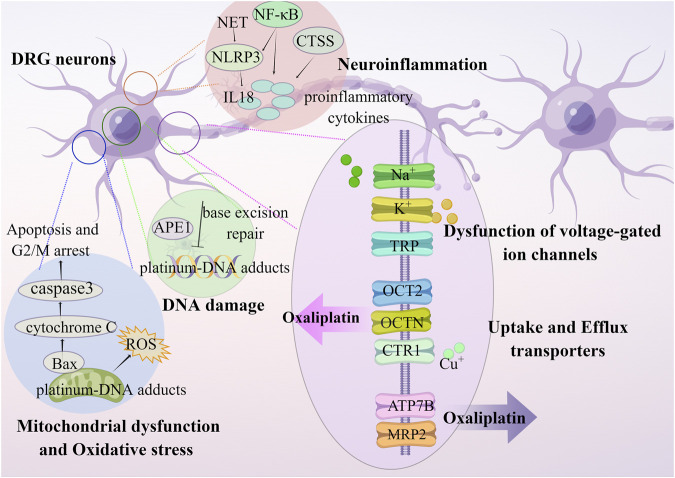
OIPN is related to mechanisms of DNA damage, dysfunction of voltage-gated ion channels, neuroinflammation, transporters, oxidative stress, and mitochondrial dysfunction.

Oxaliplatin cause apoptosis of dorsal root ganglion (DRG) neurons, but has less neurotoxic to DRG neurons due to forming fewer platinum-DNA adducts compared to cisplatin (Ta et al., 2006). The oxaliplatin-induced physical damage leads to functional impairment in neurons through DNA damage, dysfunction of voltage-gated ion channels, neuroinflammation, transporters, oxidative stress, mitochondrial dysfunction, and apoptosis (Figure 1) (Sisignano et al., 2014; Cavaletti and Marmiroli, 2020; Sałat, 2020).

### 3.1 DNA damage in sensory neurons

OPIN was considered to be secondary to DNA damage of sensory neurons, and the base excision repair pathway was the main method for improving DNA damage (Hu et al., 2019). Kelley *et al.* reported that reduction of apyrimidinic endonuclease/redox factor-1 (APE1) in neuronal cultures increased OPIN (Kelley et al., 2014), and targeting of the APE1 small molecule APX3330 and APX2009 effectively protected against OIPN without affecting the anticancer activity (Kelley et al., 2014; Kelley et al., 2016). Moreover, both thymidylate synthase and the excision cross-complementing expression were predictive markers of OIPN sensitivity (Shirota et al., 2001; Arnould et al., 2003).

### 3.2 Dysfunction of voltage-gated ion channels

An ever-growing number of studies have shown that oxaliplatin increased cold sensation through regulating the transcription of different ionic conductances (Na^+^ channels, K^+^ channels) that together shape the response of sensory neurons to cold. Therefore, the prevention of OIPN should be based on ion channels’ protection.

Acute OIPN is associated with regulation of axonal membrane Na^+^ channels, and chronic dysfunction of sensory axonal excitability occurs with a growing accumulation of oxaliplatin. Oxaliplatin was enabled to alter the voltage-gated Na^+^ channels via a pathway entailing Ca^2+^ which was possibly fixed by its metabolite oxalate (Grolleau et al., 2001). Imbalance of Na^+^ voltage-operated channels caused transient axonal hyperexcitability, which contributed to sustained depolarization that motivated the reverse pattern of Na/Ca^+++2+^ exchanger 2, leading to toxic Ca^2+^ accumulation and axonal damage (Ballarini et al., 2022). Park *et al* assessed severity for OIPN by sensory axonal excitability techniques to identify pre-clinical nerve dysfunction (Park et al., 2009). In a recent study, the Na/H exchanger isoform-1 (NHE1) was also identified as an essential contributor to intracellular pH (pH^++^i) homeostasis in nociceptors as a plasma membrane protein (Dionisi et al., 2023). They revealed that intracellular acidification induced by oxaliplatin in DRG neurons was primarily determined by Ca^2+^/calmodulin-dependent phosphatase calcineurin-mediated NHE1 inhibition.

Oxaliplatin improved hyperexcitability by decreasing the expression of diverse K^+^ channels (TREK1 and TRAAK) and improving the expression of pro-excitatory channels (hyperpolarization-activated channels) (Descoeur et al., 2011). Sittl *et al.* indicated that flupirtine, a clinically available analgesic activating slow axonal K^+^ channels in the A-fibers of peripheral nerve, alleviated the acute OIPN by suppressing axonal hyperexcitability (Sittl et al., 2010). Thus, activation of slow K^+^ channels potentially reduces OIPN in humans. Additionally, oxaliplatin antagonized voltage-operated K^+^ channels on the peripheral myelinated nerve fibers with a similar pattern of action to that of 4-aminopyridine (a classical antagonist of voltage-operated K^+^ channels) (Kagiava et al., 2008). Preclinical data showed that riluzole inhibited both sensory and motor dysfunctions through the TREK-1 potassium channel in a mouse model of chronic OIPN (Poupon et al., 2018). Argyriou *et al.* produced evidence that the repeat polymorphism of the voltage-gated K^+^ channel KCNN3 had no effect on OIPN (Argyriou et al., 2019).

Transient receptor potential (TRP) channels are related to progression of oxaliplatin-induced neuropathic pain. Oxaliplatin-induced cold allodynia was partly involved with high expression of TRP melastatin 8 (TRPM8) in the primary afferents (Gauchan et al., 2009; Mizoguchi et al., 2016). Further animal experiments showed that oxaliplatin produced a distinct increase of TRPV1, TRPM8, and TRPA1 expression in the lumbar DRG (Chukyo et al., 2018; Miguel et al., 2022).

### 3.3 Neuroinflammation

OIPN is associated with increased pro-inflammatory responses in DRGs and peripheral nerves. As a result of neutrophil extracellular trap (NET), NLRP3 was activated, and IL18 was released, which contributed to the development of OIPN (Lin et al., 2022). Oxaliplatin-treated mice showed elevated levels of NF-κB p65 protein, pro-inflammatory cytokines, and immune cell infiltration, accompanied by loss of intraepidermal nerve fibers, mechanical hyperalgesia, and a decrease in sensory nerve amplitudes (Calls et al., 2022), all of which were effectively prevented by both minocycline and niclosamide treatment (Boyette-Davis and Dougherty, 2011; Cerles et al., 2017).

It is said that Cathepsin S (CTSS), a lysosomal cysteine protease located steadily in the cytoplasm of immune-relevant cells, facilitates the activation of microglial cells and then modulates the release of proinflammatory cytokines and chemokines. Chen *et al.* found that oxaliplatin increased CTSS expression though strengthening cytosol translocation of interferon response factor 1 (Chen et al., 2021). Thus, targeting the enzymatic activity of CTSS with pharmacological blocks and gene knockdown strategies could relieve OIPN by a mechanism related to inhibition of CTSS facilitating olfactory receptor transcription factor 1 release from P300/CBP binding and then driving IL-10 downstream signaling pathway.

### 3.4 Transporters

Transporters have been identified as crucial regulators of drug disposition, therapeutic efficacy, and adverse events, because they regulate the absorption, distribution, metabolism, and excretion of drugs (Sprowl et al., 2013a; Sprowl et al., 2016). OIPN is associated with uptake and efflux of oxaliplatin by the transporter expressed on DRG cells, such as organic cation transporter (OCT) 2, organic cation/carnitine transporters (OCTN), copper transporter 1 (CTR1), P-type ATPases, and multidrug resistance-associated protein 2 (MRP2).

Oxaliplatin was found to be a relatively good substrate for human OCT2 in the HEK293 cells transiently expressing OCTs (Yonezawa et al., 2006). The evidence was provided for the critical role of OCT2 in OIPN. Sprowl *et al.* found that cellular uptake of oxaliplatin was significantly increased in cells overexpressing mouse OCT2 or human OCT2 and was decreased by cimetidine (a known OCT2 competitive inhibitor) (Sprowl et al., 2013b). In addition, genetic and pharmacological knockouts of OCT2 prevented mice hypersensitivity to cold or mechanically-induced allodynia. Similarly, Huang *et al.* also demonstrated that targeting OCT2 with genetic and pharmacological means improved acute and chronic neurotoxicity in the satellite glial cells (Huang et al., 2020). Furthermore, Jong *et al.* reported that uptake and cytotoxicity of oxaliplatin increased in HEK293 cells overexpressing rat OCTN1, rat OCTN2, human OCTN1, and human OCTN2, and that OCTN1-mediated transport of oxaliplatin seemed to make a greater contribution to its neuronal accumulation and neurotoxicity compared to OCTN2 or OCTs (Jong et al., 2011).

Rat CTR1 (rCTR1) can transport copper and platinum drugs, and makes cells susceptible to their cytotoxicity (Liu et al., 2009; Liu et al., 2013). Interestingly, in cultured rat DRG and HEK/rCTR1 cells exposure to oxaliplatin, the accumulation of platinum was saturable and temperature-dependent, but was reduced by copper only in HEK/rCtr1 cells (Liu et al., 2013). Although CTR1 regulates cellular uptake of copper, its removal is mediated by two P-type ATPases, ATP7A and ATP7B, and ATP7B is closely related to resistance to platinum drugs through regulation of efflux (Martinez-Balibrea et al., 2009). A GEMCAD group study showed that the ATP-binding cassette subfamily G, member 2 (ABCG2) rs3114018 A/A genotypes were related to a higher risk of severe OIPN (Custodio et al., 2014). MRP2, encoded by the ABCC2 gene and highly expressed in the normal gastrointestinal system, functions as a poly-specific drug efflux pump to transport a number of substrates across cell membranes through benefiting from energy produced by ATP hydrolysis (Jemnitz et al., 2010). Overexpression of MRP2 inhibited oxaliplatin accumulation and cytotoxicity, which were reversed by suppression of MRP2 with myricetin or siRNA knockdown (Myint et al., 2019). A pharmacogenomic study reported that neurotoxicity above grade 2 was correlated with single-nucleotide polymorphisms in ABCC1 [rs2074087: odds ratio = 0.43 (0.22-0.86)], and ABCC2 [rs3740066: 2.99 (1.16-7.70); rs1885301: 3.06 (1.35-6.92); rs4148396: 4.69 (1.60-13.74); rs717620: 14.39 (1.63-127.02)] (Cecchin et al., 2013).

In conclusion, some data are available to support the function of the mentioned genetic variants of the transporter in the severity of OIPN, yet the results still need to be confirmed by appropriate comprehensive and prospective large-scale studies.

### 3.5 Oxidative stress

Oxidative stress, a core mediator of apoptosis, neuroinflammation, metabolic disorders, and bioenergetic depletion in neurons, is a vital pathogenic mechanism of OPIN (Areti et al., 2014). Oxaliplatin accumulation can lead to oxidative stress in the neurons directly by the formation of DNA adducts or indirectly by mitochondrial dysfunction of electron transport chain.

Mangafodipir, a magnetic resonance imaging contrast agent, possess SOD-, catalase-, and GSH reductase–like properties. A study was performed to suggest that mangafodipir prevented and/or alleviated OIPN in cancer patients by targeting multiple steps of the reactive oxygen species (ROS) cascade via detoxifying superoxide anions and hydrogen peroxide and via restoring GSH (Coriat et al., 2014). Calmangafodipir, originated from mangafodipir, simulates the mitochondrial enzyme manganese superoxide dismutase (MnSOD), thereby reducing ROS and protecting against OIPN without apparent influence on tumour outcomes (Karlsson et al., 2015; Glimelius et al., 2018; Canta et al., 2020).

Monosialotetrahexosylganglioside (GM1) is an effective drug for the treatment of diabetic peripheral neuropathy. GM1 decreased anti-oxidant stress by increasing superoxide dismutase and GSH levels to reduce the severity of chronic OIPN (Zhou et al., 2021). However, the phase III study of GM1 did not support the use of GM1 to prevent cumulative OIPN, although patients receiving GM1 were less disturbed by acute neuropathic symptoms (Wang et al., 2020). In addition, clinical data also suggest L-carnosine exhibited a neuroprotective activity against OIPN in CRC patients by targeting Nrf-2 and NF-κB pathways (Yehia et al., 2019).

### 3.6 Mitochondrial dysfunction

Mitochondrial dysfunction is a key factor of OIPN (Canta et al., 2015; Krukowski et al., 2015). Oxaliplatin exerted anticancer properties through crosslinks forming platinum-DNA adducts that led to inhibition of DNA synthesis, mitochondrial dyfunction and ROS production (McDonald and Windebank, 2002; Zheng et al., 2011). Xiao *et al.* indicated that additional mitochondrial dysfunction worsened the neuropathic pain (Xiao and Bennett, 2012). Oxaliplatin-induced apoptosis and G2/M arrest in colon cancer cells are mediated by the apoptotic cascade, with recruitment of Bax to mitochondria and release of cytochrome C into the cytosol, leading to activation of caspase3 (Arango et al., 2004). An animal experiment showed that OIPN was concomitant with mitochondrial swelling and vacuolation of peripheral nerve axons, and that acetyl-L-carnitine protected mitochondrial function to inhibit the development of neuropathy (Zheng et al., 2011).

## 4 Neuroprotective strategies

Risk factors for OIPN consist of dose per cycle, cumulative dose, treatment regimen, duration of infusion, administration of chemotherapeutics, comorbidity and pre-existing peripheral neuropathy (Miltenburg and Boogerd, 2014). Several strategies have been proposed to reduce or prevent OIPN, including alternating chemotherapy protocols to decrease the cumulative dose of oxaliplatin and combining with chemoprotectants.

### 4.1 Dose and schedule modification

Considering reduction of treatment duration without loss of efficacy, cost of 3-month adjuvant CAPOX appears to be a promising option for high-risk stage II colon cancer (Iveson et al., 2021).

### 4.2 Reintroduction

Oxaliplatin reintroduction might be an operational choice in patients previously having moderate or severe OIPN. Intermittent oxaliplatin had a significant benefit on both time-to-treatment failure and time-to-tumor progression, and reduction of neurotoxicity compared with continuous oxaliplatin (Tournigand et al., 2006; Hochster et al., 2014). Compared with mFOLFOX6 and bevacizumab followed by FOLFIRI plus bevacizumab in patients with mCRC, Upfront FOLFOXIRI addition of bevacizumab and reintroduction after progression had a longer median progression-free survival (19.2 months *versus* 16.4 months, respectively), with no reduction of treatment efficacy and no increase in grade 3 or 4 side effects except for a predicted higher incidence of neurotoxicity (Kato et al., 2018; Cremolini et al., 2020). Oxaliplatin reintroduction in 25 mCRC patients after previously receiving FOLFOX or XELOX worsen the pre-existing OIPN, which significantly correlated with higher oxaliplatin cumulative dose. Argyriou *et al.* provided an explanation that the majority of reintroduced patients (having a clinically significant grade 1 or 2) progressed to a clinically significant (grade 2) OIPN instead of a treatment-emergent grade 3 (Argyriou et al., 2021). Surely, neurological and hypersensitivity reactions monitoring should be considered (Besora et al., 2018; Kim et al., 2018).

### 4.3 Chronomodulated oxaliplatin infusion

Circadian rhythms lead to predictable changes in the body’s tolerance and responsiveness to drugs, including anticancer agents (Lévi, 2001). Chronotherapy, the chronomodulated infusion of oxaliplatin, 5-FU and leucovorin to treat mCRC patients, showed fewer side effects, including stomatitis and peripheral sensory neuropathy, and higher objective response, when compared to constant-rate oxaliplatin infusion (Ohdo, 2003). A study comparing the delivery of oxaliplatin by chronomodulation with constant-rate delivery was conducted by Lévi *et al.* The authors observed that chronomodulated oxaliplatin infusion was more effective and less toxic than oxaliplatin delivered at constant rate over time (Lévi et al., 1994). Severe stomatitis incidence (grade 3 and 4) was 5-fold higher in patients on constant-rate oxaliplatin, compared to those submitted to chronomodulated infusion. Peripheral sensitive neuropathy (grade 2) which was cumulative dose-limiting toxicity of chronomodulated oxaliplatin was reversible following oxaliplatin withdrawal.

### 4.4 Topical administration

CRC metastases are frequently found in the liver, lungs, and peritoneum. In this context, oxaliplatin pharmacokinetics of new drug delivery strategies (HAIC, PIPAC, and HIPEC) differed partly from these of IV. Ultrafiltrate platinum of HAIC for hepatic metastases CRC had lower Vd and comparable CL than that of intravenous infusion, possibly related to slightly reduction systemic availability and higher availability at the liver organ of the drug during HAIC than its IV. PIPAC and HIPEC are treatments for CRC patients with peritoneal metastases. Total and ultrafiltrate platinum from PIPAC at dose of 120 mg/m^2^ were 3.8% and 10.2% respectively of that reported for 2-h IV of single-dose oxaliplatin at 130 mg/m^2^, not likely to induce significant systemic adverse events. HIPEC resulted in high intratumoral oxaliplatin penetration and low concentration in plasma, thus improving local tissue concentrations and reducing systemic toxicity. HIPEC combined with cytoreductive surgery led to improve survival and lower peritoneal recurrence rates (Glehen et al., 2004). However, this review was limited by the inability to directly compare the pharmacokinetics, pharmacodynamics, and toxicity of oxaliplatin after IV administration with those after HIAC, PIPAC, and HIPEC.

### 4.5 Combination of oxaliplatin with chemoprotectants

There are no established agents recommended for the prevention of OIPN in CRC patients treated with neurotoxic agents, while duloxetine was recommended for patients with CRC experiencing OIPN (Albers et al., 2011; Hershman et al., 2014).

#### 4.5.1 GSH

Cascinu *et al.* supported that GSH is a potential candidate in the prevention of OIPN, without reducing oxaliplatin activity. The authors found that neurophysiologic investigations (sural sensory nerve conduction) demonstrated a statistically significant decrease in the placebo arm than GSH-exposed group (Cascinu et al., 2002)^.^ Milla *et al.* later found that coadministration of GSH with FOLFOX4 is an effective strategy to reduce neurotoxicity without impairing the main pharmacokinetics of oxaliplatin, nor the platinum-DNA adduct formation (Milla et al., 2009). Twenty-seven CRC patients who underwent curative resection were treated with the FOLFOX regimen. Of those, 14 patients received GSH before oxaliplatin, and 13 patients received physiological saline solution, for a maximum of 12 cycles. Upon completion of treatment, patients in the GSH arm revealed only moderate neurotoxicity with grade 1 (50%) and grade 2 (50%), whereas in the placebo arm the observed neurotoxicity was moderate to severe with grade 2 (69%) and grade 3 (31%). No grade 4 neurotoxicity was showed in any group. N-acetylcysteine, as an antioxidant thiol, enables whole blood concentration of GSH to increase, which may be protective against OIPN (Bondad et al., 2020). Overall, more studies are still needed to fully characterize the effects of GSH on OIPN in these environments.

#### 4.5.2 Ibudilast

Moreover, ibudilast, a neuroimmune modulator that slowed the progression of neurological damage (Fox et al., 2018), might be a candidate for reducing OIPN. A ‘before vs after’ study showed that reduced grade 2 neurotoxicity in 2 out of 14 patients, whereas neurotoxicity had no worsening in 12 out of participants before and after ibudilast co-treatment (Teng et al., 2020). The feasibility of co-administration of ibudilast and oxaliplatin to reduce neurotoxicity urgently needs to be evaluated in large-scale studies.

#### 4.5.3 Ca/Mg

Up to date, there is no consensus on the efficacy of Ca/Mg infusions to prevent induced neurotoxicity. Based on retrospective studies, Ca/Mg infusions inhibited the incidence and intensity of acute OIPN and might delay cumulative neuropathy (Gamelin et al., 2004; Grothey et al., 2011). Subsequently, numerous studies have questioned the benefits of Ca/Mg infusions in reducing acute OIPN (Gamelin et al., 2008; Wu et al., 2012; Han et al., 2013). Large-scale randomized, controlled clinical trials in CRC population are necessary to confirm these preliminary data.

#### 4.5.4 Carbonic anhydrase inhibitor

FDA-approved drugs (namely, topiramate and acetazolamide) that inhibit carbonic anhydrase, an enzyme associated with haemoglobin in intracellular pH homeostasis, reverted oxaliplatin-induced modulation of TRPA1 and TRPV1 in cultured DRG neurons, as well as acute cold allodynia in mice without reducing oxaliplatin-induced cytotoxicity on cancer cells, and prevented oxaliplatin-related axonal hyperexcitability (Alberti et al., 2020; Potenzieri et al., 2020).

#### 4.5.5 Serotonin–noradrenaline reuptake inhibitor

There is growing evidence that serotonin and norepinephrine reuptake inhibitors are an effective treatment for neuropathy-related pain (Saarto and Wiffen, 2007). The mechanism of duloxetine-induced analgesia is considered to be relevant to the blockade of serotonin and norepinephrine transporters. A clinical trial showed that 59% of duloxetine-treated patients reported a greater reduction in painful OIPN compared to 38% of placebo-treated patients for 5 weeks (Smith et al., 2013). Although duloxetine is the only drug recommended by the American Society of Clinical Oncology that can be used for the management of chemotherapy induced peripheral neuropathy (Loprinzi et al., 2020), this recommendation was not followed in clinical practice. An NIH Collaboratory study of claims data showed the following incidence of new analgesic prescriptions for neurotoxicity: 7.1% for gabapentin, 0.69% for pregabalin, and 0.78% for duloxetine (Gewandter et al., 2020). Another cross-sectional study showed that the major analgesic drugs used by French oncologists were pregabalin (75.8%), amitriptyline (32.7%), gabapentin (25.5%), and duloxetine (11.8%) in the treatment of neurotoxicity (Selvy et al., 2021). A comparison of clinical trial studies indicated that a 60 mg dose of duloxetine was secondary to a 150 mg dose of pregabalin in relieving neuropathic pain (Salehifar et al., 2020).

Furthermore, venlafaxine has clinical activity against OIPN, with more frequent full relief (31.3% *versus* 5.3%) (Durand et al., 2012). A single-center retrospective case-control study reported the rates of obtaining over 75% symptomatic relief for OIPN under venlafaxine treatment were 53.5, 58.3, and 45.2% in the first, second, and third visits, respectively, compared to 0, 0, and 0% in the control group (Kus et al., 2016)^.^

Additionally, animal experiments showed the potential of vortioxetine (Micov et al., 2020), milnacipran (Andoh et al., 2015), and fluoxetine (Baptista-de-Souza et al., 2014) against oxaliplatin-induced mechanical allodynia. Of these, the reduction of pain hypersensitivity by vortioxetine, a novel antidepressant, was comparable to that of duloxetine (1–15 mg/kg), which may be associated with increased levels of serotonin and norepinephrine in the brainstem of treated OIPN mice. Nonetheless, there is inadequate appropriate evidence to support the use of the above drugs for patients with established painful OIPN.

## 5 Conclusion

**FIGURE 2:**
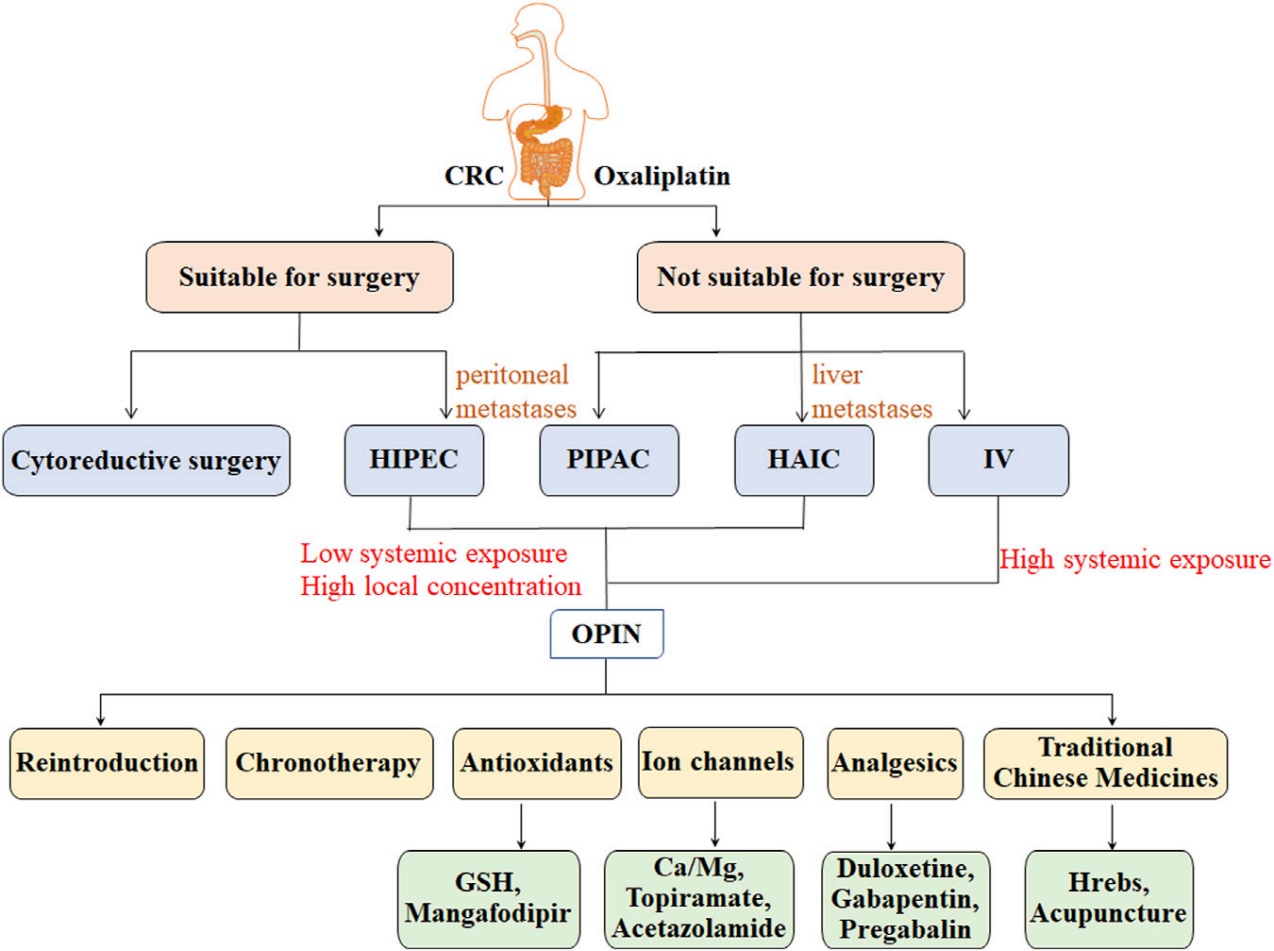
Administration routes of oxaliplatin and potential treatment options for OIPN in patients with CRC.

Ultrafiltrate platinum has an antitumor effect, at the cost of additional toxic properties. Peripheral neuropathy is recognized as a major long-term adverse effect of oxaliplatin chemotherapy, the risk of which increases due to oxaliplatin accumulation. Administration routes of oxaliplatin and potential treatment options for OIPN were shown in Figure 2.

Comparing the efficacy and OIPN of adjuvant therapy duration from 6 to 3 months in populations with different disease processes has been focused of recent studies, and patients with low-risk CRC may benefit from 3 months of CAPOX therapy (Yoshino et al., 2019; Petrelli et al., 2020; Yoshino et al., 2022). There is a positive trend towards a higher rate of organ preservation with total neoadjuvant therapy (chemoradiotherapy followed by consolidation chemotherapy, CRT-CNCT) and the watch-and-wait approach compared to induction chemotherapy followed by chemoradiotherapy (INCT-CRT) (Aref and Abdalla, 2022; Garcia-Aguilar et al., 2022; Sütcüoğlu et al., 2023). However, survival outcomes between the two TNT regimens are not different; therefore, more in-depth and rigorous studies with reliable criteria are urgently needed to explain the pros and cons of CRT-CNCT and INCT-CRT.

Topical administration (HAIC, PIPAC, and HIPEC) may be a feasible and promising strategy to increase antitumor activity while reducing neurotoxicity due to its low systemic exposure and high local concentration (Yamashita, 2004; de Jong et al., 2021). However, it is worthwhile to be alert to the risks associated with topical administration procedures, such as pump pocket complications, catheter or arterial complications, toxic or ischemic complications (Strnad et al., 2021), bowel obstruction, bleeding, abdominal pain (Alyami et al., 2019), and complications related to postoperative management (Hübner et al., 2020). Significantly, heterogeneous standardization of topical administration trials was in the context of patient selection, chemotherapy regimens, doses, number of cycles, technical protocols, and whether to combine topical administration with systemic chemotherapy, which led to controversial differences in treatment efficacy. Thus, there is an urgent need to standardize topical administration trial reports and datasets. It is suggestive for clinical practice although further validation of the effectiveness and OIPN of topical administration is required.

A *post hoc* analysis revealed difficulties in deciding the timing for discontinuation or suspension of oxaliplatin in patients with grade 2 OIPN, because physician likely underestimated OIPN via the Common Terminology Criteria for Adverse Events (CTCAE) and Functional Assessment of Cancer Therapy/Gynecologic Oncology Group-Neurotoxicity (FACT/GOG-Ntx) in patients with mCRC during early treatment (Miura et al., 2021). Perhaps, diagnostic microdosing and evaluation of multiple single nucleotide polymorphisms in oxaliplatin transporters may be a promising strategy to assess OIPN for treatment customization in CRC patients (Nichetti et al., 2019; Zimmermann et al., 2020). As a result, there is an increased need for more effective and standardized assessment methods.

An increasing number of Traditional Chinese Medicines exerted protective effects against OIPN, such as curcumin (Howells et al., 2019), forsythia viridissima (Yi et al., 2019a; Yi et al., 2019b), rutin, quercetin (Azevedo et al., 2013), Huangqi Guizhi Wuwu decoction (Cheng et al., 2017), and resveratrol (Donald et al., 2017), *etc.* Furthermore, laser acupuncture and ultrasound acupuncture significantly alleviated both oxaliplatin-induced cold and mechanical allodynia and also reduced the incidence and severity of neurotoxicity symptoms, which could be effective interventions for OIPN symptoms in patients with CRC (Hsieh et al., 2016; Chien et al., 2021).

Until now, the standard duration of adjuvant chemotherapy cycles for CRC has been between 3 and 6 months. However, efforts have been made to reduce treatment time in order to reduce toxicity. Recently developed strategies, such as chronomodulated infusion and chemoprotectants combination have been assessed to manage neurotoxicity. Further, more strategies to reduce toxicity based on pathophysiological mechanisms of neurotoxicity are necessary. Moreover, such studies should include long-term patient follow-up, and assess specific parameters such as quality of life, cost-benefit relationship, required resources, and racial disparities, among others (Kennedy et al., 2022).

## Figures and Tables

**Table:** 

**5-FU**	Fluorouracil
**APE1**	Apyrimidinic endonuclease/redox factor-1
**AUC**	Exposure/area under the curve
**CL**	Clearance
**Cmax**	Maximum concentration
**CR**	Complete response
**CRC**	Colorectal cancer
**CRT-CNCT**	Chemoradiotherapy followed by consolidation chemotherapy
**CTCAE**	Common Terminology Criteria for Adverse Events
**CTSS**	Cathepsin S
**DRG**	Dorsal root ganglion
**ePIPAC**	Electrostatic PIPAC
**FACT/GOG-Ntx**	Functional Assessment of Cancer Therapy/Gynecologic Oncology Group-Neurotoxicity
**GM1**	Monosialotetrahexosylganglioside
**GSH**	Reduced glutathione
**HAIC**	Hepatic arterial infusion chemotherapy
**HIPEC**	Hyperthermic intraperitoneal chemotherapy
**INCT-CRT**	Induction chemotherapy followed by chemoradiotherapy
**IV**	Intravenous infusion
**MATE**	Multidrug and toxin extrusion
**mCRC**	Metastatic CRC
**MRP2**	Multidrug resistance-associated protein 2
**NHE1**	Na/H exchanger isoform-1
**OCT**	Organic cation transporter
**OIPN**	Oxaliplatin-induced peripheral neurotoxicity
**PC**	Peritoneal carcinomatosis
**PD**	Progressive disease
**PIPAC**	Pressurized intraperitoneal aerosol chemotherapy
**PR**	Partial response
**RBCs**	Red blood cells
**rCTR1**	Rat copper transporter 1
**ROS**	Reactive oxygen species
**SD**	Stable disease
**t**_**1/2**_	Half-life
**TRP**	Transient receptor potential
**TRPM8**	TRP melastatin 8
**Vd**	Apparent volume of distribution
