# Supplementary material for: Oxaliplatin-induced peripheral neurotoxicity in colorectal cancer patients: mechanisms, pharmacokinetics and strategies
Source: Front Pharmacol. 2023 Aug 1;14:1231401. doi: 10.3389/fphar.2023.1231401 (PMC10427877; doi:10.3389/fphar.2023.1231401)
Supplement: Supplementary file 1 [file Table1.docx]

**Dosing and pharmacokinetic parameters of total platinum as oxaliplatin**

| **Study** | **Population (n)^a^** | **Drug time** | **Dose (mg/m^2^)** | | **Sampling**  **timepoints (n)** | **C_max_ (µg/ml)** | **AUC**  **(µg·h/ml)** | **t_½_**  **(h)** | **CL**  **(l/h)** | **V_d_**  **(l)** |  |
| --- | --- | --- | --- | --- | --- | --- | --- | --- | --- | --- | --- |
| Delord et al. ^[22]^ | | CRC (40) | IV 3 h | 130 q3w; 80/100 q2w | | 8 | NS | NS | NS | 0.127 | 17.9 |
| Merkel et al. ^[23]^ | | GIC (-/7) | IV 12 h/d 4 d | 25 q12h | | 9(day1) | 0.23 ± 0.13 | 3.9 ± 2.2 (AUC_0–24)_ | 37.7 ± 3.2 | NS | NS |
|  |  |  |  |  |  | 10(day4) | 0.50 ± 0.21 | 10.5 ± 4.5 (AUC_0–24)_ | 50.1 ± 35.3 | NS | NS |
| Gamelin et al. ^[24]^ | | CRC (16) | IV 2 h | 130 q3w | | 6 | 3.20 ± 0.61 | NS | 216±16.8 | NS | NS |
| Van et al. ^[32]^ | | mCRC (23) | IV 2 h | 130 q3w | | 12 (-erlotinib) | 3.75 ± 0.52 | 66.97 ± 16.48 (AUC_0-48_) | 33.47 ± 9.00 | NS | NS |
|  |  |  |  |  |  | 12 (+erlotinib) | 4.02 ± 0.44 | 67.45 ± 11.99 (AUC_0-48_) | 33.62 ± 7.03 | NS | NS |
| Shin et al. ^[33]^ | | mCRC (9) | IV 2 h | 130 q3w | | 8 | 4.89 ± 1.31 | 35.61 ± 6.57 (AUC_0-15_)  79.93 ± 28.59 (AUC_inf_) | 15.13 ± 5.33 | 1.81± 0.69 | 35.7 ± 7.47 |
| Kupsch et al. ^[35]^ | | refractory solid tumors (18/37) | IV 2 h | 130 q3w | | 8(-Sorafenib) | 5.09 | 66.7 (AUC_0-48)_ | NS | NS | NS |
|  |  |  |  |  |  | 8(+Sorafenib) | 6.14 | 74.7 (AUC_0-48)_ | NS | NS | NS |
| Van Cutsem et al. ^[36]^ | | mCRC (126) | IV 2 h | 85 q2w | | 8(day 1) | 2.35 | 47.9 (AUC_0-48)_ | NS | NS | NS |
|  |  |  |  |  |  | 8(day 15) | 2.29 | 48.9 (AUC_0-48)_ | NS | NS | NS |
| Schultheis et al. ^[38]^ | | mCRC (45) | IV 2 h | 85 q2w | | 13 (Cycle 1) | 2.2 | 81 | 46.4 | NS | NS |
|  |  |  |  |  |  | 13 (Cycle 2) | 2.4 | 112.9 | 52.0 | NS | NS |
| Wasserman et al. ^[39]^ | | GIC (24/39) | IV 2 h | 85 | | 10 | NS | 144 ± 37 | 123 ± 38 | 0.65 ± 0.19 | NS |
|  |  |  |  | 110 | |  | NS | 187 ± 52 | 116 ± 22 | 0.64 ± 0.20 | NS |
| Falcone et al. ^[42]^ | | mCRC (42) | IV 2 h | 100 q2w | | 11 | 2.85 ± 0.34 | 205.6 ± 38.8 | 201.4 ± 45.3 | 0.59 ± 0.15 | NS |
| Gil-Delgado et al. ^[43]^ | | CRC (34) | IV 4 h | 65 q2w | | 8 | NS | 2.62 ± 1.07 | NS | 0.48 ± 0.28 (mg/l) | NS |
|  |  |  |  | 75 q2w | |  | NS | 3.31 ± 2.04 | NS | 0.42 ± 0.24(mg/l) | NS |
| Cattel et al. ^[45]^ | | CRC(13） | IV 12 h/d 4 d | 30 q2w | | 15 (1st Course) | 0.96 | 185.01 (AUC_tot)_ | 145.9 | 1.04 | 243.9 |
|  |  |  |  |  |  | 15 (6st Course) | 1.88 | 379.22 (AUC_tot)_ | 168.9 | 0.59 | 95.6 |
| Han et al. ^[46]^ | | CRC (20) | IV 2 h | 130 | | 13 (-Ca/Mg) | 1.62 ± 0.33 | 3.30 ± 0.65 (AUC_0-5_) | NS | 34.5 ± 7.98 | 57.3 ± 14.3 |
|  |  |  |  |  |  | 13(+Ca/Mg) | 1.56 ± 0.32 | 3.08 ± 0.68 (AUC_0-5_) | NS | 36.7 ± 10.2 | 60.7 ± 17.8 |
|  |  |  |  | 85 | | 13 (-Ca/Mg) | 1.09 ± 0.14 | 2.22 ± 0.21 (AUC_0-5_) | NS | 30.7 ± 6.34 | 53.6 ± 12.5 |
|  |  |  |  |  |  | 13(+Ca/Mg) | 1.01 ± 0.10 | 2.22 ± 0.20 (AUC_0-5_) | NS | 5.19 ± 0.53 | 57.5 ± 10.3 |
| Milla et al. ^[48]^ | | CRC (27) | IV 2 h | 85 q2w | | 4(-GSH) | 2.66 | 166.95 (AUC_tot_) | 40.11 | 0.96 | 67.54 |
|  |  |  |  |  |  | 4(+GSH) | 2.47 | 127.26 (AUC_tot_) | 37.45 | 1.08 | 60.46 |
| Cho et al. ^[52]^ | | CRC (9) | IV 2 h | 130 q3w | | 5 | 4.66 ± 1.38 | 205.75 ± 53.9 (AUC_0–96)_ | 42.84 ± 10.44 | 1.03 ± 0.25 | 59 ±12 |
| Shirao et al. ^[53]^ | | CRC (9) | IV 2 h (single dose) | 130 q3w | | 12 | 3.22 ± 0.18 | 258.3±58.3 | 7.8±3.3 | 0.52±0.13 (l/h/m^2^) | 127.2±19.4（l/m^2^） |
|  |  |  |  | 90 q3w | |  | 2.26 ± 0.15 | 244.3±19.7 | 7.6±4.8 | 0.37±0.03 (l/h/m^2^) | 110.3±13.7（l/m^2^） |
| Kim et al. ^[64]^ | peritoneal metastases GIC (5/16) | PIPIC 0.5 h | 45 q6w | | 9 | 0.33 ± 0.010 | 2.77 ± 0.72 (AUC_inf)_ | 13.6 ± 2.7 | 13.54 ± 3.26 | 259.28 ± 55.49 |  |
|  |  |  | 60 q6w | |  | 0.31 ± 0.10 | 4.89 ± 5.03 (AUC_inf)_ | 16.4 ± 2.3 | 19.48 ± 16.63 | 467.70 ± 442.82 |  |
|  |  |  | 90 q6w | |  | 0.72 ± 0.39 | 7.54± 1.68 (AUC_inf)_ | 15.5 ± 2.1 | 10.27 ± 1.49 | 226.96 ± 1.13 |  |
|  |  |  | 120 q6w | |  | 1.02 ± 0.46 | 9.97 ± 4.17 (AUC_inf)_ | 18.7 ± 4.1 | 10.75 ± 3.82 | 295.67 ± 147.35 |  |
| Lurvink et al. ^[67]^ | peritoneal metastases CRC (20) | ePIPAC 0.5 h | 92 q6w | 10 (PIPAC 1) | | 2.67 | 49.0 (AUC_0_-_24)_  95.4 (AUC_0_-_48)_ | NS | NS | NS |  |
|  |  |  |  | 10 (PIPAC 2) | | 2.92 | 59.2 (AUC_0_-_24)_  111.0 (AUC_0_-_48)_ | NS | NS | NS |  |
|  |  |  |  | 10 (PIPAC 3) | | 3.28 | 59.5 (AUC_0_-_24)_  114.9 (AUC_0_-_48)_ | NS | NS | NS |  |

C_max_ maximum concentration, AUC_0-t_ exposure/area under the curve from time zero to t, t_1/2_ half-life, CL clearance, Vd apparent volume of distribution, PIPAC pressurized intraperitoneal aerosol chemotherapy, ePIPAC electrostatic PIPAC, HIPEC hyperthermic intraperitoneal chemotherapy
