# Supplementary material for: Oxaliplatin-induced peripheral neurotoxicity in colorectal cancer patients: mechanisms, pharmacokinetics and strategies
Source: Front Pharmacol. 2023 Aug 1;14:1231401. doi: 10.3389/fphar.2023.1231401 (PMC10427877; doi:10.3389/fphar.2023.1231401)
Supplement: Supplementary file 2 [file Table2.docx]

**Dosing and pharmacokinetic parameters of ultrafiltrate platinum as oxaliplatin**

| **Study** | **Population (n)a** | **Drug time** | **Dose (mg/m^2^)** | **Sampling timepoints (n)** | | **Cmax (µg/ml)** | **AUC**  **(µg·h/ml)** | **t½**  **(h)** | **CL**  **(l/h)** | **Vd**  **(l)** |
| --- | --- | --- | --- | --- | --- | --- | --- | --- | --- | --- |
| Delord et al. ^[22]^ | CRC (40) | IV 3h | 130 q3w;  80 q2w; 100 q2w | | 8 | NS | NS | NS | 18.7 | 40.8 |
| Merkel et al. ^[23]^ | GIC (-/7) | IV 12-h/d 4 d | 25 q12h | | 9(day1)  10(day4) | 0.08 ± 0.01 | 1.0 ± 0.3 (AUC_0–24)_ | 12.6 ± 4.2 | NS | NS |
|  |  |  |  | |  | 0.13 ± 0.002 | 1.9 ± 0.4 (AUC_0–24)_ | 15.5 ± 7.0 | NS | NS |
| Gamelin et al. ^[24]^ | CRC (16) | IV 2 h | 130 q3w | | 6 | 0.45±0.075 | NS | 171.6±16.8 | NS | NS |
| Van et al. ^[32]^ | MCRC (23) | IV 2 h | 130 q3w | | 12 (-erlotinib) | 0.60 ± 0.17 | 7.50 ± 1.49 (AUC_0-48_) | 14.50 ± 2.69 | NS | NS |
|  |  |  |  |  | 12 (+erlotinib) | 0.58 ± 0.19 | 7.32 ± 1.25 (AUC_0-48_) | 16.05 ± 3.91 | NS | NS |
| Shin et al. ^[33]^ | MCRC (9) | IV 2 h | 130 q3w | | 8 | 1.52 ± 1.41 | 5.23 ± 2.55 (AUC_0-15_)  9.25 ±1.35 (AUC_inf)_ | 13.25 ± 8.21 | 14.32 ± 2.41 | 256.68±138.99 |
| Kupsh et al. ^[35]^ | refractory solid tumors (18/37) | IV 2 h | 130 q3w | | 8(-Sorafenib) | 2.45 | 6.94 (AUC_0-48)_ | NS | NS | NS |
|  |  |  |  |  | 8(+Sorafenib) | 1.90 | 6.47 (AUC_0-48)_ | NS | NS | NS |
| Schultheis et al. ^[38]^ | mCRC (45) | IV 2 h | 85 q2w | | 13(Cycle 1) | 0.8 | 4.2 | 17.2 | NS | NS |
|  |  |  |  |  | 13 (Cycle 2) | 1.0 | 4.9 | 19.0 | NS | NS |
| Wasserman et al. ^[39]^ | GIC (24/39) | IV 2 h | 85 | | 10 | NS | 7.9 ± 4.5 | 23.2 ± 8.8 | 14.8 ± 11.1 | NS |
|  |  |  | 110 | |  | NS | 9.2 ± 2.6 | 25.5 ± 7.7 | 12.8 ± 3.7 | NS |
| ForNSro et al. ^[40]^ | mCRC (15) | IV 2 h | 85 | | 11 | 1.17 ± 0.21 | 12.5 ± 3.9 | NS | 7.43 ± 2.82 | NS |
| Kemeny et al. ^[41]^ | mCRC (49) | IV 2 h | 60 qw | | 7 (+irinotecan) | NS | NS | 31.7 ± 34.2 | 19.1 ± 10 | 552 ± 243 |
| Falcone et al. ^[42]^ | mCRC (42) | IV 2 h | 100 q2w | | 11 | 0.82 ± 0.09 | 10.91 ± 1.82 | 198.3 ± 29.4 | 10.12 ± 2.33 | NS |
| Gil-Delgado et al. ^[43]^ | CRC (34) | IV 4h | 65 | | 8 | NS | 0.12 ± 0.04 | NS | 10.29 ± 6.32(mg/l) | NS |
|  |  |  | 75 | |  | NS | 0.11 ± 0.07 | NS | 13.32 ± 8.8(mg/l) | NS |
| Pfeiffer et al. ^[44]^ | CRC (70) | IV 0.5h | 130 q3w | | 10 | 5.76 ± 0.59 | 3.45 ± 0.33 | 0.24 | 36.5 ± 3.8 | 0.29 ± 0.04 |
| Cattel et al. ^[45]^ | CRC(13） | IV 12h/d 4 d | 30 q2w | | 15 (1st Course) | 0.14 | 866.91 (AUC_tot)_ | 38.3 | 25.8 | 1601.6 |
|  |  |  |  |  | 15 (6st Course) | 0.17 | 19.50 (AUC_tot)_ | 73.8 | 10.3 | 1270.9 |
| Han et al. ^[46]^ | CRC (20) | IV 2 h | 130 | | 13 (-Ca/Mg) | 1.92 ± 0.21 | 4.25 ± 0.60 (AUC_0-5_) | NS | 26.8 ± 6.57 | 49.7 ± 11.7 |
|  |  |  |  |  | 13(+Ca/Mg) | 1.95 ± 0.26 | 4.15 ± 0.87 (AUC_0-5_) | NS | 27.8 ± 6.01 | 52.2 ± 9.82 |
|  |  |  | 85 | | 13 (-Ca/Mg) | 1.43 ± 0.30 | 2.98 ± 0.80 (AUC_0-5_) | NS | 22.9 ± 6.89 | 43.9 ± 9.17 |
|  |  |  |  |  | 13(+Ca/Mg) | 1.54 ± 0.26 | 2.93 ± 0.54 (AUC_0-5_) | NS | 23.3 ± 5.17 | 46.4 ± 10.4 |
| Teng et al. ^[47]^ | GIC (12/16) | IV 2 h | 130/85 | | 5(-Ibudilast) | 0.014 (ug/l)/(mg) | 0.111 (AUC_0-24)_ (ug.h/l)/(mg)_)_ | NS | 3.96 | NS |
|  |  |  |  |  | 5(+Ibudilast) | 0.016 (ug/l)/(mg) | 0.112 (AUC_0-24_ (ug.h/l)/(mg)_)_ | NS | 3.90 | NS |
| Milla et al. ^[48]^ | CRC (27) | IV 2 h | 85 q2w | | 4(-GSH) | 0.20 | 4.43 (AUC_tot_) | 24.01 | 33.21 | 1320.6 |
|  |  |  |  |  | 4(+GSH) | 0.20 | 4.49 (AUC_tot_) | 19.31 | 31.06 | 822.40 |
| Cho et al. ^[52]^ | CRC (9) | IV 2 h | 130 q3w | | 5 | 0.84 ± 0.13 | 30.05±8.23 (AUC_0–96)_ | 29.17±5.88 | 7.2 ± 2.2 | 225 ±71 |
| Shirao et al. ^[53]^ | CRC (9) | IV 2 h  (single dose) | 130 q3w | | 12 | 1.45±1.67 | 11.3±1.5 | 258.9 ± 61.4 | 11.7 ± 1.4 (l/h/ m^2^) | 1612.7±360.5（l/m^2^） |
|  |  |  | 90 q3w | |  | 0.96±0.10 | 7.9 ± 0.9 | 246.7 ± 32.2 | 11.6 ± 1.4 (l/h/ m^2^) | 1433.4±196.3（l/m^2^） |
| Kern et al. ^[56]^ | CRC (13) | IV 4h | 130 q3w | | 17 | 1.61 ± 0.55 | 20.17 ± 6.97 | 27.3 ± 10.6 | 13.32 ± 3.9 | 349 ± 132 |
| Kern et al. ^[61]^ | Hepatic metastases CRC (21) | HAIC 4 h | 135 q3w | | 17 | NS | 17.76 ± 7.8 | 17.75 ± 9.29 | 8.13 ± 3.51 | 10.66 ± 6.71 |
| Kim et al. ^[64]^ | peritoneal metastases GIC (5/16) | PIPIC 0.5 h | 45 | | 9 | 0.02 ± 0.004 | 0.32 ± 0.12 (AUC_inf)_ | 17.1 ± 5.6 | 49.44 ± 15.64 | 1130.76 ± 223.96 |
|  |  |  | 60 | |  | 0.02 ± 0.005 | 0.46 ± 0.30 (AUC_inf)_ | 16.6± 4.4 | 56.72 ± 42.88 | 1194.51 ± 697.07 |
|  |  |  | 90 | |  | 0.04 ± 0.03 | 0.59 ± 0.11 (AUC_inf)_ | 15.9 ± 3.2 | 51.76 ± 5.12 | 1181.09 ± 193.44 |
|  |  |  | 120 | |  | 0.11 ± 0.06 | 1.15 ± 0.23 (AUC_inf)_ | 18.2 ± 4.3 | 34.30 ± 5.07 | 888.61 ± 148.76 |
| Dumont et al. ^[65]^ | peritoneal metastases GIC (5/10) | PIPAC 0.5 h | 90 | | 8 | 0.90 | 6.11(AUC_0_-_24)_  6.78(AUC_0_-_∞)_ | 15.2 | 12.4 | NS |
|  |  |  | 140 | | 8 | 1.04 | 9.03(AUC_0_-_24)_  10.09(AUC_0_-_∞)_ | 13.6 | 13.0 | NS |
| Lurvink et al. ^[67]^ | peritoneal metastases CRC (20) | ePIPAC 0.5 h | 92  q6w | | 10 (PIPAC 1) | 1.36 | 9.6 (AUC_0_-_24)_ | NS | NS | NS |
|  |  |  |  |  | 10 (PIPAC 2) | 1.38 | 11.7(AUC_0_-_24)_ | NS | NS | NS |
|  |  |  |  |  | 10 (PIPAC 3) | 1.90 | 11.7(AUC_0_-_24)_ | NS | NS | NS |
| Elias et al. ^[69]^ | peritoneal carcinomatosis (6/20） | HIPEC 0.5 h | 460 | | 14 | 13.2 | 14.8 ± 3.8 | NS | NS | NS |

C_max_ maximum concentration, AUC_0-t_ exposure/area under the curve from time zero to t, t_1/2_ half-life, CL clearance, Vd apparent volume of distribution, PIPAC pressurized intraperitoneal aerosol chemotherapy, ePIPAC electrostatic PIPAC, HIPEC hyperthermic intraperitoneal chemotherapy
